# Effect of Solvothermal Temperature on Morphology and Supercapacitor Performance of Ni-MOF

**DOI:** 10.3390/molecules27238226

**Published:** 2022-11-25

**Authors:** Wanxin Shen, Xiaotian Guo, Huan Pang

**Affiliations:** 1 School of Chemistry and Chemical Engineering, Yangzhou University, Yangzhou 225009, China; 2 Interdisciplinary Materials Research Center, Institute for Advanced Study, Chengdu University, Chengdu 610106, China

**Keywords:** MOF, temperature, supercapacitor

## Abstract

A series of Ni-MOF materials were synthesized via a simple hydrothermal method and can be employed as electrodes for supercapacitors (SCs). Different temperatures were selected to unveil the effect of temperature on the formation, structure, and electrochemical performance of Ni-MOF-x (x = 60, 80, 100, and 120). Ni-MOF-80 possessed a larger specific surface area with a cross-network structure formed on its surface. The synthesized Ni-MOF electrode delivered a specific capacity of 30.89 mA h g^−1^ when the current density reached 1 A g^−1^ in a three-electrode system. The as-fabricated Ni-MOF materials could be further designed and are expected to deliver satisfactory performance in practice.

## 1. Introduction

Metal–organic frameworks (MOFs) are material formed by strong bonds connecting transition metal ions and organic ligands with periodic network construction. Therefore, MOFs can be fabricated into a variety of products, with different geometry and dimensions, by changing the collocation of the metal ions and linkers This material has excellent strength because of its structure porosity and large specific area [1,2,3]. MOF materials have been applied in various fields dealing with gas adsorption, seawater desalination, and biomedicine [4,5,6,7]. Furthermore, MOF materials have revealed great potential in electric energy storage applications, including batteries and SCs [8].

Supercapacitors (SCs) have attracted wide attention as a type of electrochemical energy storing device since the 1990s [9,10,11,12]. Combining the advantages of batteries and conventional capacitors, SCs could fill the gap between the above two energy storage devices. In addition to energy and power density, the service life is also an important criterion to determine the performance of an energy storage system. SCs have higher charge and discharge rates and a longer cycling life because of the non-Faraday mechanism. Therefore, materials with a larger specific surface area have the potential to be made into electrode sheets. Conventional electrode raw materials contain transition metal oxides, conducting polymers, and carbon-based materials [13,14,15,16]. The mentioned materials have both advantages and disadvantages. Carbon materials are faced with the problem of low energy density, and conductive polymers often suffer from expanding and disintegrating during the charge–discharge reaction, and metal oxides have poor conductivity and a relatively expensive price [17]. As a multifunctional energy storage material, MOFs can be applied to the application of SCs [18,19,20], and they are an attractive material for pseudo-capacity because of their large number of available metal redox sites, large surface area, adjustable aperture, and scaffold structure [21,22].

There are many choices of metal sources for MOF materials, such as Mo, Fe, Co, Ni, and Cu [23,24,25,26,27]. Among them, Ni-MOF has attracted much attention in electrochemical energy storage applications containing lithium-ion batteries (LIBs) and SCs due to its great redox ability. As a member of the transition metals, nickel can offer redox-active sites and own different oxidation states. When participating in redox reactions, Ni-MOFs can be more active than other MOFs and show excellent electrochemical properties. Therefore, Ni-MOFs as supercapacitor electrodes have shown considerable prospects [28,29,30]. Two-dimensional (2D) and three-dimensional (3D) micro-/nanosized MOFs have received much attention for supercapacitor applications because of their large specific surface areas and low electrical resistance [31,32,33,34,35]. Cuboid Ni-MOF [36], nanoflower-like Ni-MOF [37], accordion-like Ni-MOF [38], and nanosheet-assembled Ni-MOF micro flowers [32] have been prepared through a facile hydrothermal method. For example, Wang et al. synthesized a novel moss-like 3D Ni-MOF self-assembled by numerous nanorods with different thicknesses and lengths. The 3D Ni-MOF had a maximum specific capacity of 638 C g^−1^ at 0.5 A g^−1^ [39]. Kale obtained a nanoflower-like Ni-MOF with a high specific capacity of 467 C g^−1^ at 1 A g^−1^ in an aqueous 6 M KOH electrolyte [37]. The hydrothermal reaction time played a critical role in the morphology and structure of Ni-MOF [40]. Manikandan et al. fabricated Ni-MOF by a hydrothermal method with a reaction time of 30 h. The obtained product exhibited a high specific capacitance of 1498.6 F g^−1^ at 1 A g^−1^ [41].

The electrochemical performance of SC is related to the morphology or structure and composition of the electrode materials [42,43]. Synthetic conditions containing elemental metals, organic linkers, molar ratios, solvents, and technological parameters can control the morphology and porosity of the MOF [44,45]. The solvothermal method is a synthetic way that the powder of reactants is dissolved in a solvent and recrystallized in a sealed pressure vessel. The phase, size, and shape of the product can be well controlled by this method. In the synthesis process of MOF, in addition to the above conditions, the temperature in the solvothermal method plays an important role. Supramolecular isomerism is quite easy to be formed for central metal ions with flexible and diverse coordination patterns and organic ligands with multiple coordination sites. Any reaction requires a certain temperature to obtain energy and overcome the energy barrier, and fabricating MOF is no exception. Secondly, the properties of the solvent will change at different temperatures; for example, the solubility of the reactants varies at different temperatures [46,47,48,49]. The reaction temperature was adjusted quite often when synthesizing the MOF compounds by the hydrothermal or solvothermal synthesis methods.

In this work, we systematically studied the influence of temperature on the synthesis process of Ni-MOF. The experiment results indicate that solvothermal temperature can significantly influence the structure and properties of the produced MOF. The product synthesized at 60 °C exhibited a hexahedral morphology, and fragments were formed on the surface. When the solvothermal temperature was 80 °C, the cross-linked network construction appeared on the surface of hexahedral Ni-MOF clearly. The surface of Ni-MOF obtained at 100 °C became smooth and formed a compact structure. According to the three-electrode test system, the Ni-MOF-80 showed the best performance, which could be attributed to the excellent morphology and structure. The Ni-MOF-80 with a unique cross-linked morphology structure had an abundant hierarchical interspace and a high specific surface area, which were beneficial to the transport of electrons and ions and provided more exposed active sites to achieve an enhanced electrochemical performance as electrode material. In addition, this novel porous structure also had considerable potential in catalysis and gas adsorption.

## 2. Results and Discussion

**Figure 1:**
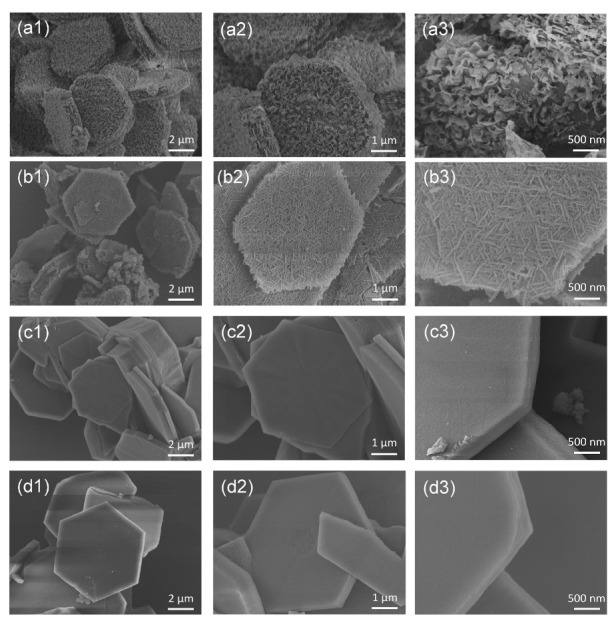
SEM images of Ni-MOF produced at different reaction temperatures, (**a1**–**a3**) Ni-MOF-60; (**b1**–**b3**) Ni-MOF-80; (**c1**–**c3**) Ni-MOF-100, and (**d1**–**d3**) Ni-MOF-120.

A series of Ni-MOF materials were synthesized at different temperatures by a facile hydrothermal method. Correspondingly, the samples Ni-MOF-x (x represents a hydrothermal temperature) were named as Ni-MOF-60, Ni-MOF-80, Ni-MOF-100, and Ni-MOF-120 due to the reaction temperature. The microstructure of the products was amplified under scanning electron microscope (SEM). In Figure 1, the fabricated Ni-MOF is a hexagonal construction with an average thickness of about 500 nm. After further magnification, it could be clearly seen that the surface of Ni-MOF appears as a cross-linked network construction. According to Figure S1, the whole growth process of hexagonal Ni-MOF was formed by a gradual stacking of sheets by controlling the dosages of reactants.

**Figure 2:**
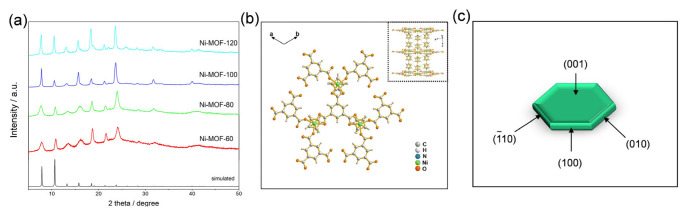
(**a**) X-ray diffraction patterns of Ni-MOF-60, Ni-MOF-80, Ni-MOF-100, and Ni-MOF-120; (**b**) Schematic diagram of Ni-MOF, Ni: green, C: gray, O: orange, and N: blue; (**c**) Diagram of MOF single-crystal figure.

After comparing the XRD spectrums of Ni-MOFs in Figure 2a, the patterns of as-synthesized samples and reference samples were consistent, indicating that the crystal structures of the samples were the same [50]. The synthesized hexagonal Ni-MOF presents a 3D-layered structure in Figure 2b. The 2D planar layer is composed of divalent nickel ion and partially deprotonated H_3_BTC. 4,4′-bipyridine acts as the ligand to connect the 2D layer to form a 3D porous skeleton construction. The specific morphology of Ni-MOF can be observed from Figure 2c and Figure S2. The top and bottom hexagonal planes were the (001) crystal plane, and the other planes were the (010), ($\bar{1}$10), and (100) crystal planes. The (001) crystal plane was the largest revealed surface, which helped to speed up the electron transport and diffusion of the electrolyte solution, so that the 4,4′-bipyridine–nickel chain connected to each 3D plane created conductive paths for the electrons perpendicular to the (001) crystal plane. According to Figure 1b, the cross-linked porous network structures were also formed on the crystal planes. These structures optimized the diffusion and storage of the electrolyte solution, which improved the performance of the electrode materials in the SC systems. The N_2_ adsorption/desorption isotherms and the pore size distribution curves of a series of Ni-MOFs are shown in Figure S6. The obtained materials showed an obvious combination of Type I and Type IV isotherms. The curves increased sharply at low pressures, almost reaching the horizontal plateau, which verified the presence of micropores (Type I). The presence of a hysteresis loop at higher pressures confirmed the presence of mesopores. Such an isotherm shape was designated as Type IV, corresponding to the presence of some mesopores in the microporous solids. The specific surface areas of Ni-MOFs are summarized in Table S1; Ni-MOF-80 showed the largest specific surface area up to 612 m^2^/g.

**Figure 3:**
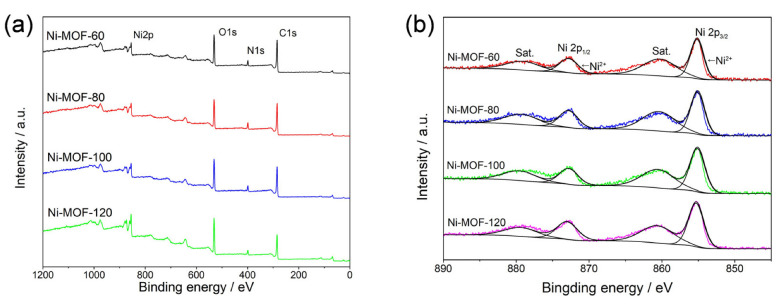
(**a**) Full XPS spectra and (**b**) Ni spectra of Ni-MOF-60, Ni-MOF-80, Ni-MOF-100, and Ni-MOF-120.

According to Figure 3a, the products consisted of Ni, O, N, and C elements by analyzing the XPS spectrum of these samples. In the case of Ni-MOF-60, four peaks can be seen in the Ni 2p spectrum in Figure 3b, and the principal spin orbital peaks are at binding energies of 872.8 and 855.2 eV, and the locations of the satellite peaks are at binding energies of 879.2 and 860.5 eV. The XPS spectrum indicates that the nickel element mainly existed in the form of a bivalent in the produced Ni-MOF. The valence of Ni of the Ni-MOFs formed at different temperatures did not shift significantly. The fitted C 1s, N 1s, and O 1s spectra are shown in Figures S3–S5. For Ni-MOF, the peaks located at 288.2, 285.3, and 284.5 eV are related to the O–C=O, C–O, and C=C–C bonds resulting from the organic ligands (4,4′-Bipy and H_3_BTC) [51]. The fitted N 1s are shown in Figure S4. The binding energies of the peaks are 399.4 and 398.8 eV arising from N-Ni and bipyridine-N [52].

Then, the properties of the product as a working electrode were studied. The CV curves of all samples in Figure S7 clearly showed a pair of redox peaks, which revealed that the Faraday behavior of the electrode appeared in the reaction. As the scanning speed accelerated, the peak current and curve area both increased. The above phenomenon indicated that the electrode showed excellent electrochemical reversibility and charge storage performance. It was the redox reaction of nickel in different valence states in the SC system that led to the appearance of this Faraday feature. In addition, the redox reaction of the following equation could be used to describe the charge storage mechanism:

Ni(II)_s_ + OH^−^ ↔ Ni(II)(OH)_ad_ + e^−^ (1)

Ni(II)(OH)_ad_ ↔ Ni(III)(OH)_s_ + e^−^ (2)

Figure S8 shows the GCD curves of the four samples from 1.0 A g^−1^ to 5.0 A g^−1^. The charge–discharge voltage recorded during the electrode reaction showed a similar linear relationship with the increase in time, which all showed pseudo-capacity behavior. As the current density gradually increased, the observed charge–discharge curves still revealed a symmetrical curve pattern, and each curve had a typical discharge platform because of the redox reaction in the test process. When the current applied to the electrodes increased, the surrounding active substances attracted abundant ions. Therefore, the ion concentration at the interface between the active substance and the electrolyte decreased. Then, the diffusion velocity of the ions in the electrolyte was lower than the charging and discharging speeds. As a result, the electrode liquid diffused through the electrode interface resulting in a polarization effect. The capacity of the electrodes obviously decayed at a high current density.

**Figure 4:**
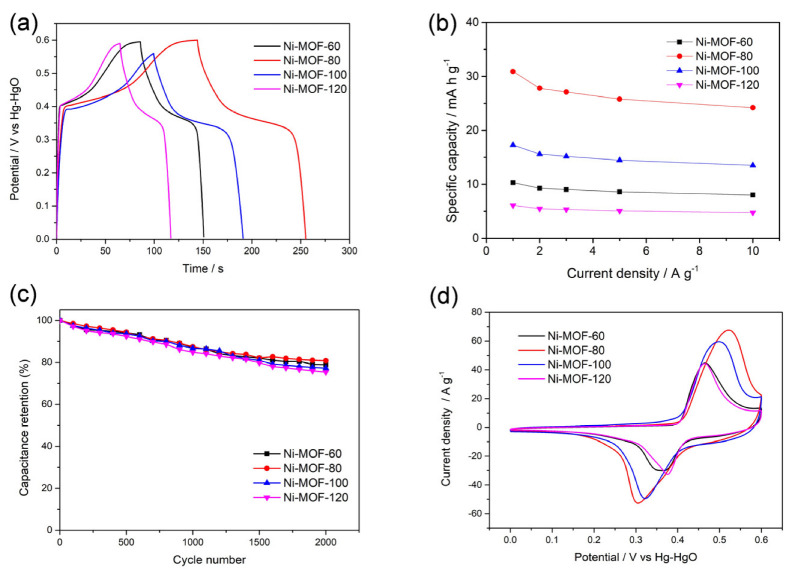
(**a**) GCD drawings of different Ni-MOFs at 1 A g^−1^; (**b**) Specific capacity of four samples within the range of 1.0−10.0 A g^−1^; (**c**) Capacity retention performance of four samples; (**d**) CV curves of different Ni-MOFs at 100 mV s^−1^.

The performance of Ni-MOFs can be observed in Figure 4a. Compared with other samples, Ni-MOF-80 had the highest surface area and could be used more efficiently to accommodate large amounts of electric charge to obtain a higher capacity. Then, the specific capacity (*Cs*, mA h g^−1^) of the electrode can be calculated in terms of the following equation:

(3) $$C_{S}=\frac{2I\times \int V\;dt}{m\times ∆V}$$

I (mA) represents the current density; ∫V dt is on behalf of the integrated area under the discharge curve; m (g) represents the load mass of the sample coated on the electrode, and ∆V (V) displays the value of the voltage window [53,54]. The specific rate performance of the electrode itself can be observed and analyzed through the obtained results. The electrochemical properties of the Ni-MOF electrodes at the current density in the range of 1.0–10.0 A g^−1^ are shown Figure 4b. The charge storage capacities of Ni-MOF-80 were 30.89, 27.83, 27.09, 25.83, and 24.17 mA h g^−1^ at different current densities. The specific capacity of the electrode was inversely proportional to the current density. When the current intensity increased, the specific capacity of the electrode decreased. The cycling stable ability of the prepared samples was revealed by the GCD endurance test; when the current density was 5 A g^−1^ (Figure 4c), the capacity of all Ni-MOF electrodes remained above 75% after 2000 cycles, indicating that the electrodes had good electrochemical reversibility in the charging and discharging processes. Among them, Ni-MOF-80 still had the best performance and maintained the highest capacity. Electrochemical impedance spectroscopy (EIS) was carried out to further investigate the electron transfer and ion diffusion at the electrode/electrolyte interface, and the Nyquist plot is shown in Figure S9. The Ni-MOF-80 nanocomposites had preferably steep slopes in the low-frequency region of the Nyquist plots, which indicated that Ni-MOF-80 showed faster ion transportation and electrolyte diffusion. Comparing various MOF-based electrode materials for the SC applications (Table S2), it was noted that the supercapacitor performance of the Ni-MOF-80 needed to be improved in future. The as-obtained material had great potential with its excellent structure.

## 3. Materials and Methods

Nickel (II) nitrate hexahydrate (Ni(NO_3_)_2_·6H_2_O, 0.1455 g, 0.5 mmol), trimesic acid (H_3_BTC, 0.105 g, 0.5 mmol), and 4,4-Bipyridine (4,4′-bipy, 0.0780 g, 0.5 mmol) were dissolved in 10 mL *N, N*-Dimethylformamide (DMF) and then thoroughly stirred at room temperature until the solution became clarified. Then, the products were synthesized by a solvothermal method at 60 °C, 80 °C, 100 °C, and 120 °C for 24 h in drying oven. After the metal shells had cooled to room temperature, the obtained green products in Teflon liners were absterged by DMF, ethanol, and deionized water. In the end, the obtained samples were put in vacuum oven at 50 °C and kept for 24 h. The products were marked according to the reaction temperature.

A CHI760E electrochemical workstation was utilized for recording electrochemical performance, via cyclic voltammetry, galvanostatic charge–discharge, and electrochemical impedance spectroscopy of fabricated Ni-MOF in conventional three-electrode system at 3 M KOH electrolyte. The three-electrode system consisted of as-synthesized Ni-MOF-coated Ni foam, Hg/HgO electrode and platinum wire as working electrode, reference electrode, and auxiliary electrode, respectively. The loading mass of Ni-MOF on nickel foam was around 2 mg, and efficient surface area was around 1 × 1 cm^2^. CV curves at different scanning rates (5–100 mV s^−1^) were obtained at a constant voltage. GCD curves were measured by changing the current density from 1.0 to 5.0 A g^−1^ at a constant voltage. EIS spectra were measured at frequencies ranging from 0.1 Hz to 100 kHz.

## 4. Conclusions

In this work, we adopted an easy solvothermal method to fabricate a series of Ni-MOF materials by controlling the reaction temperatures. The morphologies of the Ni-MOF materials synthesized under different solvothermal temperatures were closely related with the electrochemical property. The cross-linked networking structure was generated on the surface at 80 °C. The Ni-MOF-80 with a larger specific surface area showed a better supercapacitor performance than other samples. When worked in the three-electrode system at the current density of 1 A g^−1^, Ni-MOF-80 delivered a specific capacity of 30.89 mA h g^−1^. Further, the cross-linked hexagonal Ni-MOF-based materials can be expected to be used in other applications, such as efficient gas separation, water treatment, hydrogen evolution reaction, etc.

## Data Availability

Data are contained within the article or Supplementary Materials.

## Supplementary Materials

The following supporting information can be downloaded at: https://www.mdpi.com/article/10.3390/molecules27238226/s1. Figure S1: SEM images of samples fabricated at different dosages of reagents; Figure S2: The related plane images of the Ni-MOF; Figure S3: C 1s spectra of Ni-MOF-60, Ni-MOF-80, Ni-MOF-100, and Ni-MOF-120; Figure S4: N 1s spectra of Ni-MOF-60, Ni-MOF-80, Ni-MOF-100, and Ni-MOF-120; Figure S5: O 1s spectra of Ni-MOF-60, Ni-MOF-80, Ni-MOF-100, and Ni-MOF-120; Figure S6: BET curves and the pore size distribution curves of a series of Ni-MOF, (**a**) Ni-MOF-60, (**b**) Ni-MOF-80, (**c**) Ni-MOF-100, and (**d**) Ni-MOF-120; Figure S7: CV curves of a series of Ni-MOF in three-electrode system, (**a**) Ni-MOF-60, (**b**) Ni-MOF-80, (**c**) Ni-MOF-100, and (**d**) Ni-MOF-120; Figure S8: GCD curves of a series of Ni-MOF in three-electrode system, (**a**) Ni-MOF-60, (**b**) Ni-MOF-80, (**c**) Ni-MOF-100, and (**d**) Ni-MOF-120; Figure S9: Comparative EIS images of Ni-MOF-60, Ni-MOF-80, Ni-MOF-100, and Ni-MOF-120; Table S1: BET surface of Ni-MOF-60, Ni-MOF-80, Ni-MOF-100, and Ni-MOF-120,; Table S2: Various MOF materials for supercapacitor application reported in previous work. Notably, the references [55,56,57,58,59,60,61,62,63] are available for Table S2 in the Supplementary Materials.

## Sample Availability

Samples of the compounds are available from the authors.
