# Supplementary material for: Effect of Solvothermal Temperature on Morphology and Supercapacitor Performance of Ni-MOF
Source: Molecules. 2022 Nov 25;27(23):8226. doi: 10.3390/molecules27238226 (PMC9740099; doi:10.3390/molecules27238226)
Supplement: Supplementary file 1 [file molecules-27-08226-s001.zip › molecules-2028623-supplementary.pdf]

Effect of solvothermal temperature on morphology and supercapacitor performance of Ni-MOF

Wanxin Shen[1], Xiaotian Guo[1, 2, *] and Huan Pang[1, *]

1 School of Chemistry and Chemical Engineering, Yangzhou University, Yangzhou 225009, PR China. E-mail: Shenwanxin1994@163.com (W.S.); guoxiaotianchem@163.com (X.G.); huanpangchem@hotmail.com (H.P.).

2 Interdisciplinary Materials Research Center, Institute for Advanced Study, Chengdu University, Chengdu 610106, PR China.

* Correspondence: School of Chemistry and Chemical Engineering, Yangzhou University, Yangzhou, 225009, Jiangsu, PR China.

Materials and Methods

Materials and reagents:

AR grade of nickel(II) nitrate hexahydrate ($Ni(NO_3)_2·6H_2O$), 1,3,5-tricarboxlic acid (H_3BTC), 4,4-Bipyridine, N,N-Dimethylformamide (DMF) and potassium hydroxide (KOH) were purchased from Sinopharm Chemical Reagent Co., Ltd. The purchased chemicals were utilized without additional purification. DMF was used for all the solution preparation.

Methods:

Material Characterization:

The SEM morphology was studied by field emission scanning electron microscope with Gemini SEM 300 (Carl Zeiss AG). A series of the products were tested by X-ray diffraction (XRD) on a Bruker D8 Advanced X-ray Diffractometer (Cu-Kα radiation: λ=0.15406 nm) for the phase analysis. X-ray photoelectron spectroscopy (XPS) tests were conducted on a Thermo Scientific ESCALAB 250 apparatus. Additionally, Brunauer-Emmett-Teller (BET) and Barrett-Joyner-Halenda (BJH) tests were conducted on the Autosorb IQ3 instrument.

Preparation of working electrode:

Ni-MOF and acetylene black (conductive agent) were mixed with weight ratio (85:10). The above powders were mixed with polytetrafluoroethylene (PTFE) (0.05 mL) and isopropanol (10 mL) to form a homogeneous slurry. Subsequently, the suspensions (effective active area: 1 cm ×1 cm) were coated on Ni foam (1 cm×6 cm), and the coated Ni foam was got pressed at 10 MPa.

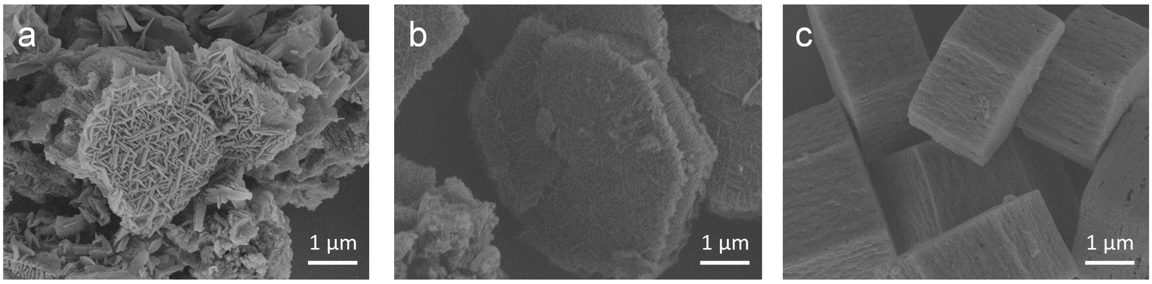

Figure S1. SEM images of samples fabricated at different dosages of reagents.

When the amount of reactant was 0.25 mmol, it could be found that the obtained product was composed of thin sheets. When the dosage was doubled up to 0.50 mmol, the sheets gradually piled up to form a compact hexagonal plate, which showed a tightly packed network structure on the surface. When the dosage was doubled again, the surface structure of the sample was tight and orderly.

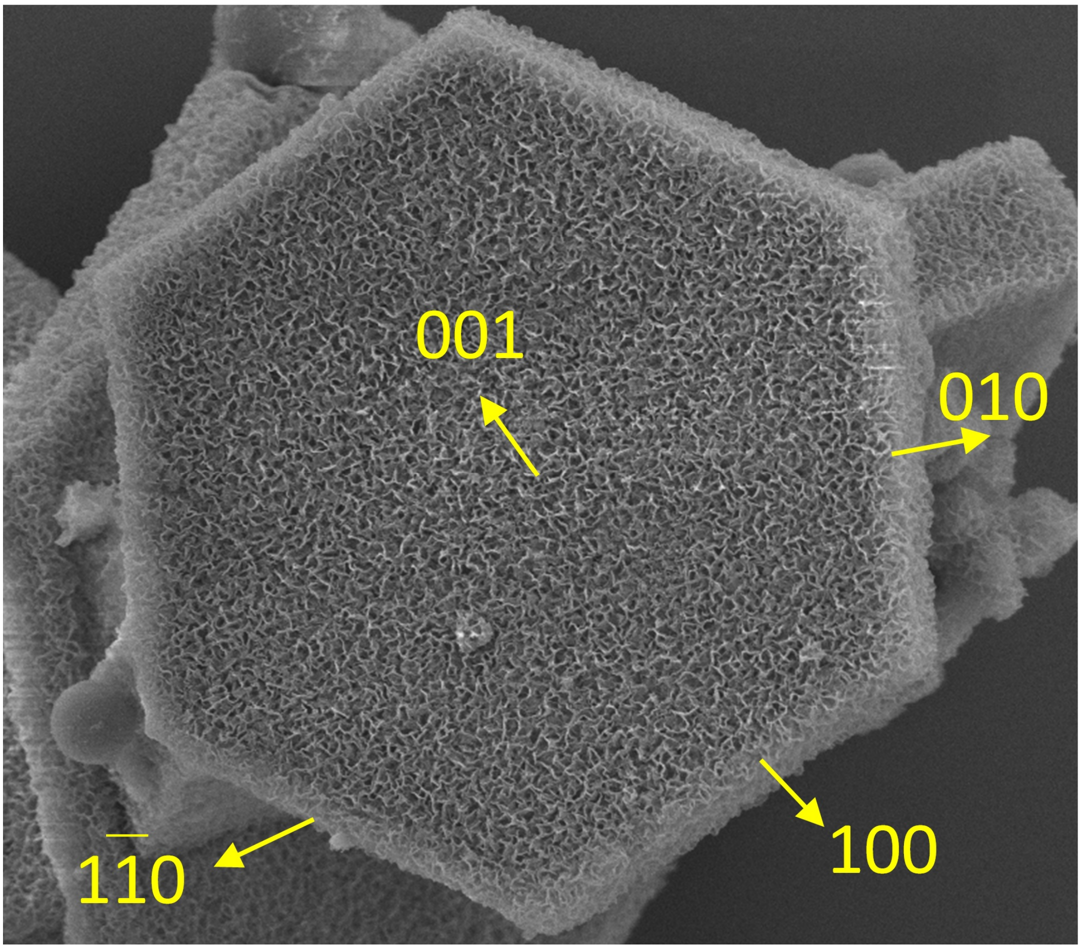

Figure S2. The related planes image of the Ni-MOF.

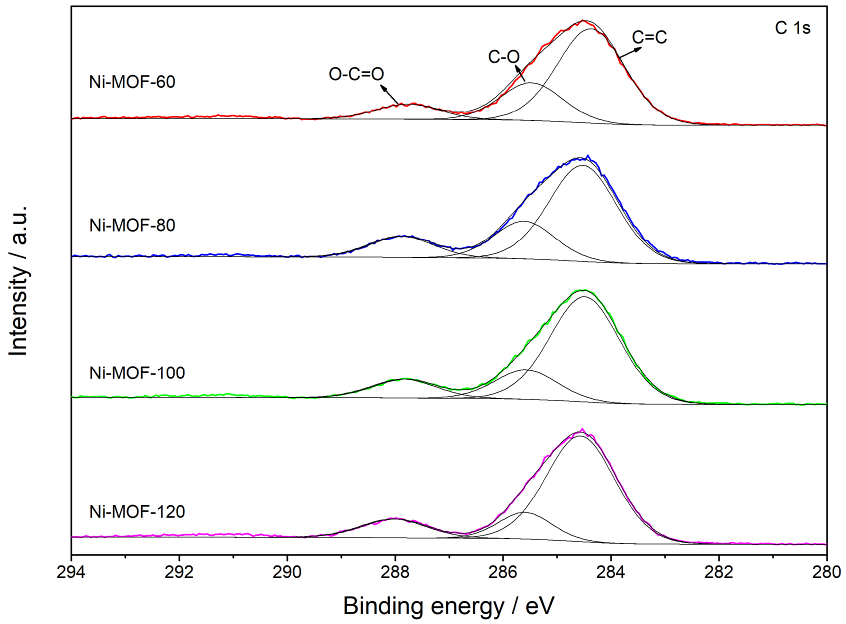

Figure S3. C1s spectra of Ni-MOF-60, Ni-MOF-80, Ni-MOF-100 and Ni-MOF-120.

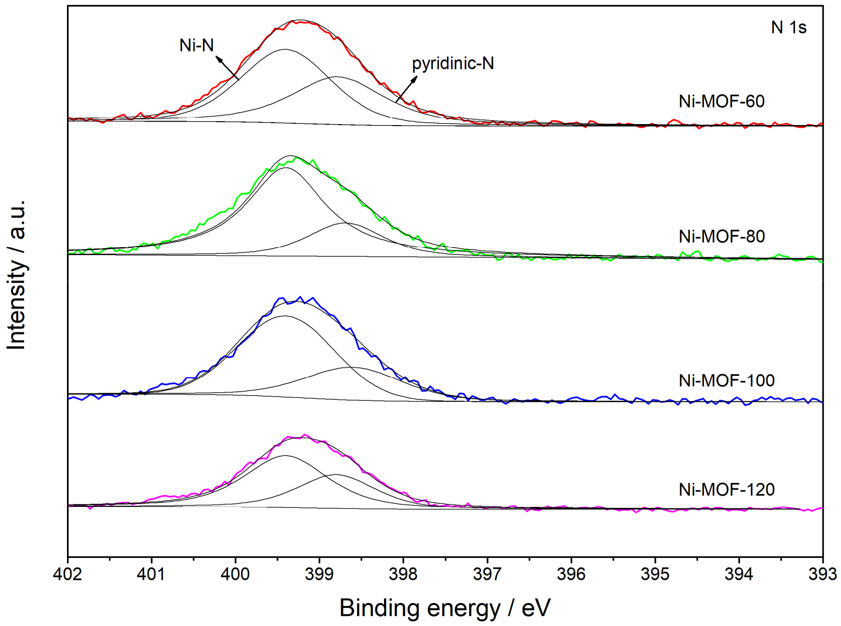

Figure S4. N1s spectra of Ni-MOF-60, Ni-MOF-80, Ni-MOF-100 and Ni-MOF-120.

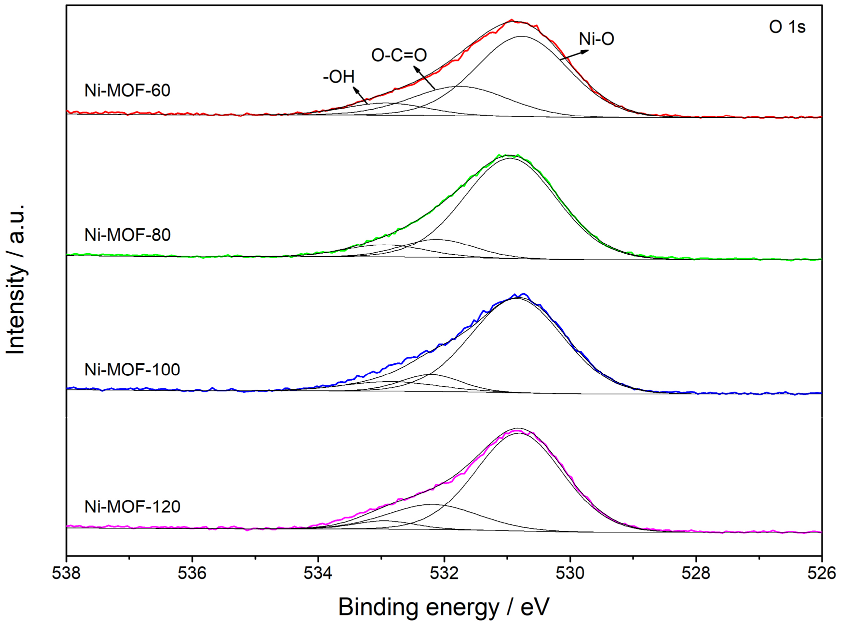

Figure S5. O1s spectra of Ni-MOF-60, Ni-MOF-80, Ni-MOF-100 and Ni-MOF-120.

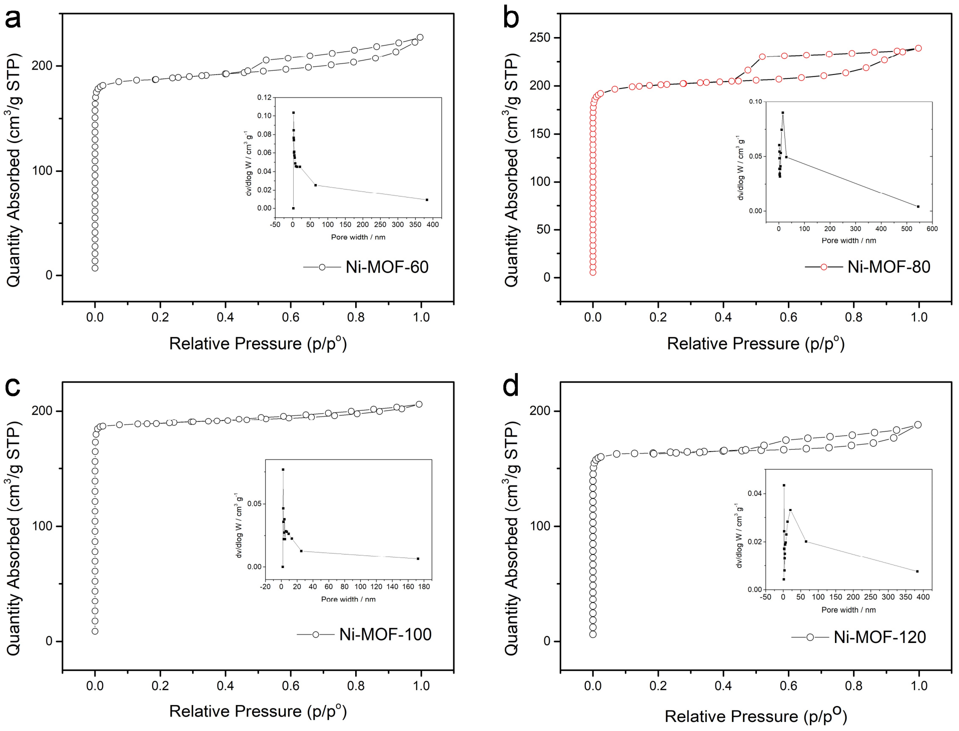

Figure S6. BET curves and the pore size distribution curves of a series of Ni-MOF, (**a**) Ni-MOF-60, (**b**) Ni-MOF-80, (**c**) Ni-MOF-100 and (**d**) Ni-MOF-120.

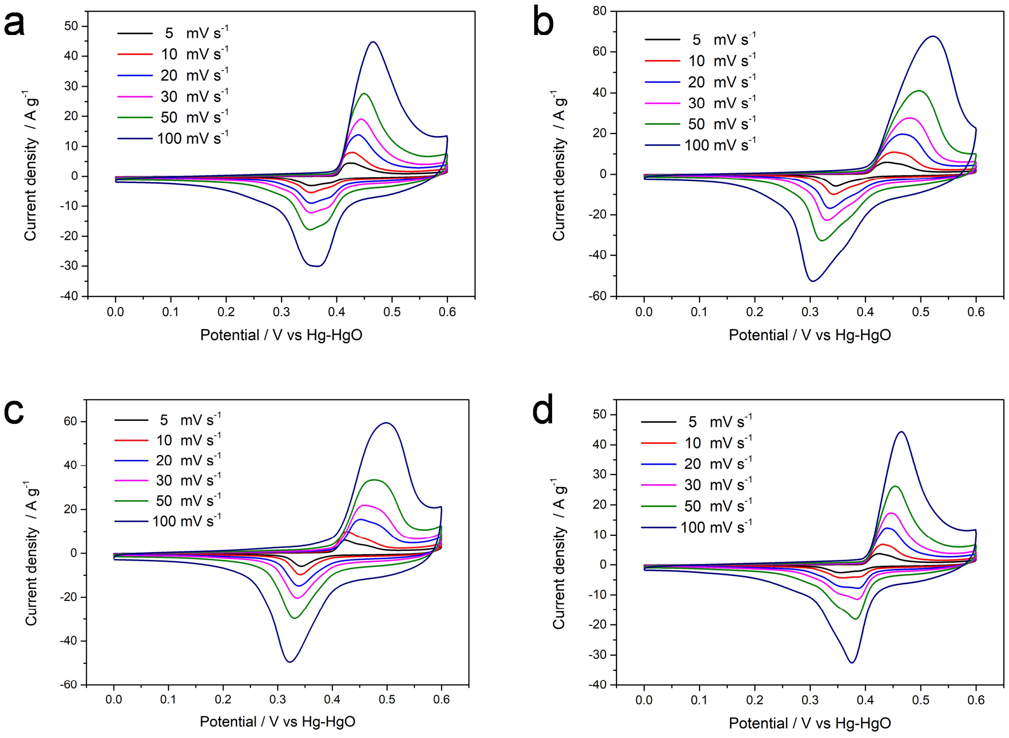

Figure S7. CV curves of a series of Ni-MOF in three-electrode system, (**a**) Ni-MOF-60, (**b**) Ni-MOF-80, (**c**) Ni-MOF-100 and (**d**) Ni-MOF-120.

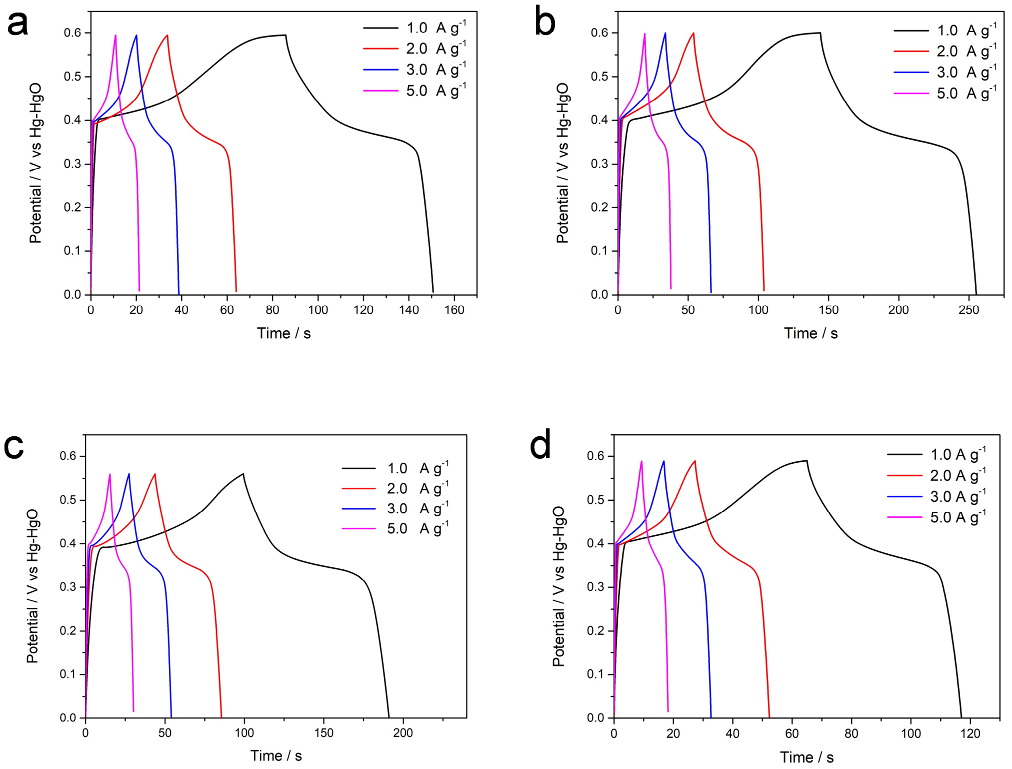

Figure S8. GCD curves of a series of Ni-MOF in three-electrode system, (**a**) Ni-MOF-60, (**b**) Ni-MOF-80, (**c**) Ni-MOF-100 and (**d**) Ni-MOF-120.

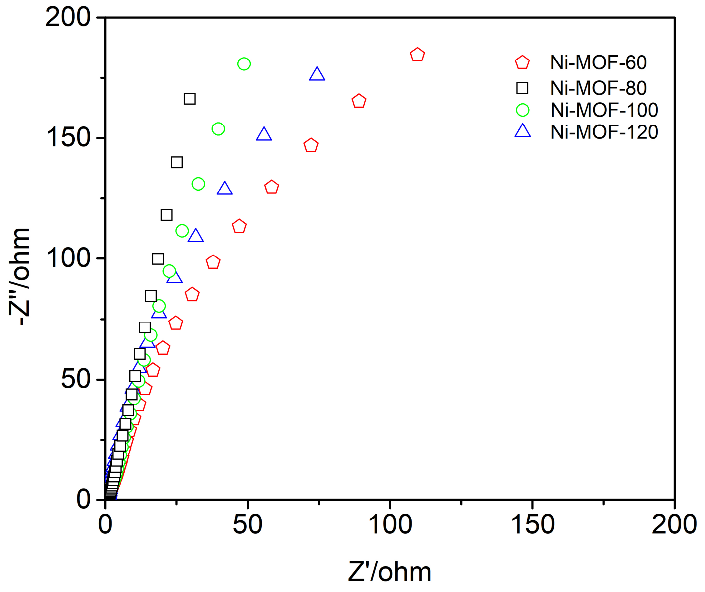

Figure S9. Comparative EIS images of Ni-MOF-60, Ni-MOF-80, Ni-MOF-100 and Ni-MOF-120.

Table S1. BET surface of Ni-MOF-60, Ni-MOF-80, Ni-MOF-100, and Ni-MOF-120, respectively.

Sample	BET surface (m^2/g)
Ni-MOF-60	564
Ni-MOF-80	612
Ni-MOF-100	559
Ni-MOF-120	488

Table S2. Various MOF materials for supercapacitor application reported in previous work.

MOF	Linker	Morphology	Electrolyte	Specific capacitance F g^{-1}	Current density	Cyclic stability
Ni-MOF [31]	H_2ppza	2D layered MOF	KOH	184	1 A g^{-1}	65% (1000)
Ni-MOF [36]	H_2bdc	Stacked layer-cuboid	2M KOH	804	1 A g^{-1}	56% (5000)
Ni-MOF [55]	4,4'-bipy	Double-ligand	3 M KOH	318	1 A g^{-1}	essentially unchanged
Ni-MOF [56]	PTA	–	3 M KOH	606.8	0.5 A g^{-1}	–
Ni-MOF [57]	H_2BDC	–	6 M KOH	356	10 mV s^{-1}	–
Co-MOF [58]	3,5-H_2pdc	–	3 M KOH	90	0.5 A g^{-1}	–
Co-MOF_{6h} [59]	H3nbta	Crystalline block	6 M KOH	450.89	0.5 A g^{-1}	95% (1000)
Cu-MOF [29]	H_3BTC	–	KOH	195	10 mV s^{-1}	–
Cu-MOF [60]	H_3BTC	Microrod structure	3M KOH	228	1.5 A g^{-1}	89.7% (3000)
CNTs@Mn-MOF [61]	$(NH_4)_2C_8H_4O_4$	–	1 M Na_2SO_4	203.1	1 A g^{-1}	88% (3000)
Mn-MOF [62]	H_3BTC	Nanorod	6 M KOH	371	0.5 A g^{-1}	91.8% (10000)
Zn-functionalized Ni-MOF [63]	–	–	3 M KOH	391	1 A g^{-1}	85% (5000)
Ni-MOF (This work)	H_3BTC 4,4'-bipy	hexagonal structure	3 M KOH	185	1 A g^{-1}	75% (2000)
